# Enhancement of Tech-Sil25 Maxillofacial Silicone Mechanical Properties after Artificial Weathering through Addition of Nanoparticles

**DOI:** 10.1155/2022/4082168

**Published:** 2022-12-31

**Authors:** Shahad Fadhil Bunyan, Fatanah Mohamad Suhaimi, Faraedon Mohidden Mostafa Zardawi, Siti Noor Fazliah Mohd Noor, Muhammad Azrul Zabidi

**Affiliations:** ^1^Department of Dental Science, Advanced Medical and Dental Institute, Universiti Sains Malaysia, Bertam, 13200 Kepala Batas, Pulau Pinang, Malaysia; ^2^Kirkuk Technical Institute, Northern Technical University, Kirkuk, Iraq; ^3^Dental Simulation and Virtual Learning Research Excellence Consortium, Advanced Medical and Dental Institute, Universiti Sains Malaysia, Penang, Malaysia; ^4^Department of Periodontics, College of Dentistry, Sulaimani University, Sulaimani, Iraq; ^5^Dean of the Faculty of Dentistry, Qaiwan International University, Sulaimania, Iraq

## Abstract

**Purpose:**

To evaluate the effect of nanosilica and nanoalumina addition in Tech-sil25 maxillofacial silicone before and after exposure to artificial weathering conditions.

**Materials and Methods:**

A total of 144 samples were divided into four groups, a control group (*n* = 12) and three test groups, nanosilica (NS) (*n* = 36), nanoalumina (NA) (*n* = 36), and a hybrid nanoparticle (HySA) (*n* = 60) at different weight percentages (1, 2, and 3 wt. %) was added to Tech-sil25. Samples were exposed to artificial weathering for 100 hours, and subjected to characterizations involving tear strength, shore A hardness, roughness, and tensile strength tests. The data were analyzed using descriptive and inferential statistics using a one-way ANOVA test to determine the level of significance between the groups.

**Results:**

After 100 hours of artificial weathering, the one-way ANOVA result shows a highly significant increase in tensile and tear strengths with a minimal increase in hardness and roughness observed in samples containing 2% nanosilica (NS) followed by hybrid nanoparticle (HySA) of 1% nanoalumina (NA) + 1% nanosilica (NS) compared with a control group and other groups.

**Conclusions:**

The addition of nanosilica (NS), nanoalumina (NA), and a hybrid nanoparticle (HySA) to the Tech-sil25 maxillofacial silicone improved its mechanical properties. The combination of several filler reinforcements is essential for enhancing silicone's antiaging properties of silicone and maintaining some of its mechanical properties to prolong the service life.

## 1. Introduction

Facial injuries include damaged facial regions caused by trauma, and facial parts congenital missing make a persistent need for maxillofacial rehabilitation [1]. Reconstruction of facial deformities can be achieved either surgically by plastic vascular surgery, which is the most desirable for the patient, or by artificial reconstruction using alloplastic materials, which are noninvasive and risk-free choices [2]. A wide variety of materials was applied throughout history for the fabrication of maxillofacial prostheses, but all materials used previously were far from the ideal material properties [3].

Soft and flexible material for maxillofacial prosthesis was described by Bulbulian in 1941 [4]. The most significant change in the history of the maxillofacial prosthesis was the invention of specific silicone rubber for the creation of facial soft tissue prostheses, which was a great challenge in the field of craniofacial reconstruction [5, 6]. As far silicone polymers are the most widely used, owing to their promising properties such as chemical stability, simplicity of production, biocompatibility, and durability. However, the material has not fulfilled the ideal properties of the maxillofacial prostheses due to some drawbacks, particularly in mechanical properties [7]. Optimization of maxillofacial materials properties is necessary to extend the service life of the prostheses. Additionally, when silicone ages, there may be visible physical and chemical changes because of a photo-oxidative attack, where the combined effects of oxygen and sunlight are key causes of deterioration while the prosthesis is being used [8].

Consequently, different methods have been suggested and applied to improve the clinical properties of silicone elastomers. Among them was the addition of fillers which would increase the material's elasticity and improve its properties both physically and mechanically, making it more practical clinically [8]. Several studies tested the effect of adding a variety of nanoparticles to the silicone polymer on the mechanical and optical properties after adding and after exposing the product to natural and accelerated weathering conditions [9].

Due to the nanoparticles' tiny size, high specific area, quantum effect, and strong interfacial interaction with the organic polymer, the addition of titanium dioxide (TiO2) and zinc oxide (ZnO) can enhance the mechanical and optical properties of polymers [10]. A systematic review by Sonnahalli and Chowdhary recommended adding nanoparticles (NPs) to silicone elastomers at different concentrations to improve the mechanical and physical properties of the prostheses, protect the prosthesis color from fading, and keep them stable during service life [11].

A significant number of research has improved the importance of the size and surface area of all nanoparticles and microfillers to enhance the properties of polymers [10]. The improvement in the mechanical properties of silicone depends on the size of filler particles, surface area, structure, and the surface activity of the fillers, as well as on polymer properties and the concentration of the filler when added, which can be changed to reach the desirable maxillofacial mechanical properties [12]. There is no ideal maxillofacial silicone material, some with good characteristics and others with poor properties. Research is still being conducted to improve the properties of silicone elastomers [13].

In this study, the null hypothesis (adding the nanoparticles to the silicone elastomer will not improve the mechanical properties) will be investigated by testing the mechanical properties of the maxillofacial silicone with the addition of nanosilica (NS), nanoalumina (NA), and a hybrid nanoparticle (HySA) by several weight percentages after subjecting to artificial weathering.

## 2. Materials and Methods

Maxillofacial silicone used in this study was the Tech-sil25 room temperature vulcanized one, a two parts silicone (Technovent Ltd., Bridgend, UK). According to the manufacturer's instructions, the mixing ratio of the base to the catalyst was 10 : 1. The mixing of the control group began with the addition of the base to the electronic balance container. Then, the catalyst was added and started mixing by the vacuum mixer with a speed of 360 rpm and a vacuum of (−10 bar). For the reinforced groups, nanopowder was added to the electronic balance container followed by the addition of the base, then mixed without vacuum for 3 minutes, followed by a vacuum for 7 minutes. Next, the catalyst was added and mixed with a vacuum for the remaining 5 minutes [14].

The nanofiller selected in this study were nanosilica (NS) and nanoalumina (NA) due to their excellent physical and mechanical properties and because they have a high surface area with small particle size and chemical activity; also, it is abundant and inexpensive. Furthermore, they are the most widely used fillers in the maxillofacial silicone elastomers, and they provide a high degree of reinforcement to silicone products [14, 15].

For each test, molds were made in accordance with the guidelines. Hardness test in accordance with ASTM D2240, 2010 standards, surface roughness test in accordance with ISO 7619-1 2010 specifications, tear strength test in accordance with ASTM D624, 2012 type C standards, and tensile strength test in accordance with ISO specification number 37 : 2011 [16]. The silicone was then poured into the molds. Then, the cover was tightened with the parts of the remaining molds by the G-clamps. After 24 hours of complete vulcanization, the RTV silicone was subjected to artificial weathering for 100 hours.

Two types of nanopowder used in this study were nanosilica (NS) oxide filler (7631-86-9) with 99.9% purity and particle size of 30–50 nm (Platonic Nanotech, Indiana, USA) and nanoalumina (NA) oxide filler (1330–060920) with 99.9% purity and particle size of 40–60 nm (SkySpring Nanomaterials Inc, USA). The mixing ratio of Tech-sil25 silicone will be 10 : 1; for the base (part A) and catalyst (part B). In this study, the amount of base mixed with nanosilica (NS), nanoalumina (NA), and a hybrid nanoparticle (HySA) is shown in Table 1.

Four main groups of 144 samples were prepared, 12 samples for the control without addition group, 36 samples for the nanosilica (NS) group, at (1%, 2%, and 3%) by weight with 12 samples for each concentration. Similarly, 36 samples were prepared for nanoalumina (NA) groups at (1%, 2%, and 3%) by weight with 12 samples for each concentration, and 60 samples for a group that hybrid nanoparticle (HySA) at (0.5% NS + 0.5% NA, 1% NS + 1% NA, 2% NS + 1% NA, 1% NS + 2% NA, and 1.5% NS + 1.5% NA) by weight with 12 samples for each concentration.

The samples were tested for tear strength, tensile strength, shore A hardness, and roughness. Plastic molds were fabricated using CNC (Computer Numerical Control) machine. Each mold consists of the base, frame, and cover parts in the exact dimensions. Type 2 dumbbell shapes were fabricated for tensile strength. Specimens were mounted on the computerized universal testing machine (25 ± 0.5 mm) (WDW-20, Laryee Technology Co. Ltd., China). A digital caliper was used to measure the sample's thickness in its center and on each end in accordance with ISO 37 : 2011 specifications and the mean of the measurements was then calculated. The samples were marked symmetrically around 10 mm from each end. After the samples broke, the maximum force reading was taken. Samples were stretched at a rate of 500 mm/min. The machine software estimated the maximum load, and the tensile strength was then computed using the following equation:

(1) $$\begin{array}{c} Ts\left[MPa\right]={\left(\frac{F}{A}\right).}^{16}\text{,} \end{array}$$

where Ts is the tensile strength (MPa); *F* is the force magnitude prior to breaking (N); *A* is the cross-sectional area of the unstrained specimen (mm^2^).

For the tear strength test, samples with flat ends and a right angle at the middle were prepared. A universal testing device (WDW-20, Laryee Technology Co. Ltd., China) was used to test the specimens at a crosshead speed of 500 mm per minute. The specimens were created in accordance with ASTM D624 (2012) for the tear strength test, as stated in ISO 37 : 2011. The machine software computed the maximum load, and the tear strength was estimated using the following formula [16]:

(2) $$\begin{array}{c} T\left[\frac{kN}{m}\right]={\left(\frac{F}{D}\right).}^{16}\text{,} \end{array}$$

where *T* is the tear strength (N/mm); *F* is the maximum force (N); *D* is the thickness of the specimen (mm).

For the shore A hardness test, specimens were fabricated according to ISO 7619-1:2010 with the dimension of 25 mm × 25 mm × 6 mm (thickness). One point was placed in the middle and four others were placed at each corner of the outside surface, with a 6 mm gap between each mark. A Shore A hardness durometer was used to take the measurements. The durometer's indenter pierced the sample surface at the five indicated locations. The mean of six measurements was recorded after the durometer was firmly depressed for three seconds. The identical shore A hardness samples were utilized for the surface roughness test, measuring 25 mm × 25 mm × 6 mm. It was measured with a profilometer tester, and the average of six readings was calculated [17].

Statistical analysis implemented using data from the experimental groups was collected and compared to the control group using one-way analysis of variance (ANOVA) for tensile strength, tear strength, surface roughness, and shore A hardness. When significant differences were observed, the Scheffe test was conducted as a post hoc test to identify differences among the groups at a significance level of *α* = 0.05 for all tests. *P* values < 0.05 were considered statistically significant. All statistical tests were conducted using the statistical software SPSS (Statistical Package for Social Sciences, version 23.0, SPSS Inc., Chicago, IL, USA).

## 3. Results

The result of the ANOVA test for silicone elastomer mechanical properties after reinforcement with nanoalumina (NA) at 1%, 2%, and 3% by weight is illustrated in Table 2. The average values for roughness and hardness increased significantly across all groups compared to the control group. For tensile strength and tear strength, the mean values of 1% and 2% NA addition were higher than the control, while the mean value was decreased with the addition of 3% NA. Hardness resulted in being the highest in 3% wt. Additionally, 2% by weight addition of NA produced the highest tear strength. The same was valid with the tensile strength cause 2% NA was the highest mean value. While the mean value gained from 3% NA was the highest for the roughness test.

Table 3 shows the descriptive statistical value and one-way analysis of variance (ANOVA) for reinforcement with nanosilica (NS) groups at 1%, 2%, and 3% by weight. The hardness mean value was higher for all addition compared to the control group. Additionally, for tensile and tear, the mean values of 2% NS were higher than the control group. The mean value for roughness was increased after adding 2% NS and 3% NS by weight compared with the control group, while it lowered at 1% NS compared with the control group. The mean value gained from 3% NA was the highest for the roughness.

The ANOVA test result of silicone elastomer mechanical properties after reinforcement with hybrid nanoparticle (HySA) groups at several wt.% of NA and NS is illustrated in Table 4. All hybrid nanoparticles (HySA) showed significantly increased mean values for hardness which was the highest in NA 1% + NS 2%, compared with the control except NA 0.5% + NS 0.5%. For tensile and tear, only two combinations NA 0.5% + NS 0.5% and NA 1% + NS 1% produced higher mean values, while the highest tear strength was NA 1% + NS 1%. The same hybrid nanoparticle resulted in the highest tensile strength based on the highest mean value compared with the control. As for roughness, all hybrid nanoparticles resulted in higher mean values except NA 0.5% + NS 0.5%, while the mean value gained from NA 2% + NS 1% resulted in the highest roughness.

Further analysis using the regression method conducted on the HySA group indicated that the increase in hardness and roughness were muchly contributed by the percentage of alumina rather than silica. The effect on hardness depends on the percentage of alumina by a factor of 3.98 versus 2.68 for silica. In contrast, roughness is subjected to alumina by 0.25 and silica by 0.15. Additionally, the tear is negatively affected by alumina with a factor of 0.69 and silica with a factor of 0.29. On the other hand, silica is indirectly proportional to the tensile with 0.39, while alumina with 0.02.

## 4. Discussion

Manufacturers typically state the mechanical value of silicones without including any colors, fillers, or additives that may not accurately reflect the material's clinical performance when used for extra-oral prostheses. As a result, while employing a material to create facial prostheses, maxillofacial prosthodontists and anaplastologists should be careful about the manufacturer's values [1].

The concept of making a composite from the combination of nanoparticles (NPs) with polymeric reinforcement as fillers and polymer as a matrix in a new three-phase composite reinforcement is a very successful composite and is used in this study. Besides, combining two different microparticles in a precise ratio could improve the overall qualities of the silicone polymers and render lifelike maxillofacial prostheses closer to the ideal properties. Thus, two types of additives were selected and used in this study in different sizes and characteristics. The additives were nanoparticles (NPs) with a high modulus of elasticity and microfillers with good flexibility [13].

The synergistic effects of different filler combinations can be explained by silicon dioxide having a small size particle in the range of 5–25 nm, a large surface area, and function activity with a robust interfacial reaction with other materials [11]. This leads to a more excellent interface between the Al_2_O_3_-SiO_2_ and silicone matrix that may explain in some instances the improvement in some of the silicone's mechanical properties [11].

**Figure 1:**
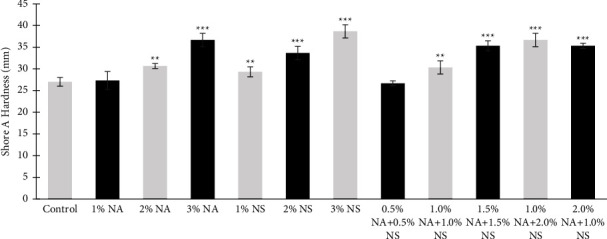
Shore A hardness for different wt. % of nanoalumina (NA), nanosilica (NS), and hybrid nanoparticle HySA.  ^*∗∗*^*P*  <  0.01,  ^*∗∗∗*^*P*  <  0.001.

The results of Shore A hardness indicated that hardness increased when the addition of nanomaterials increased for each addition of nanoparticles. The highest increase was with 3% NS (38.6) compared to the control group (27). For NA and NS hybrid nanoparticles, the highest mean for hardness was with addition NA 1% + NS 2% (36.6) compared to the control group, as seen in Figure 1. This could be a result of space-filling by nanofillers around the dispersion of microfillers that increase the stiffness of the material. Also, there is a nonsignificant increase in hardness with 1% NA which could be due to the insufficient amount of nanofillers within the material [14].

**Figure 2:**
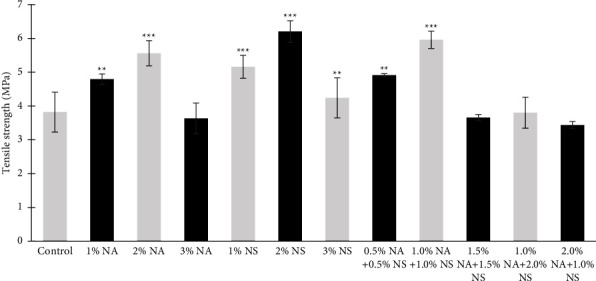
Tensile strength for different wt. % of nanoalumina (NA), nanosilica (NS), and hybrid nanoparticle HySA.  ^*∗∗*^*P*  <  0.01,  ^*∗∗∗*^*P*  <  0.001.

**Figure 3:**
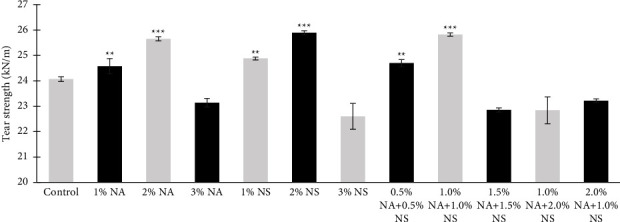
Tear strength for different wt. % of nanoalumina (NA), nanosilica (NS), and hybrid nanoparticle HySA.  ^*∗∗*^*P*  <  0.01,  ^*∗∗∗*^*P*  <  0.001.

The highest mean value for tear strength and tensile strength of maxillofacial silicone after reinforcement was 2% NS for the nanosilica group (Figure 2). Similarly, 2% NA resulted in the highest tear strength and tensile strength among the nanoalumina group (Figure 3), followed by 1% NA + 1% NS hybrid nanoparticle in Figures 2 and 3, which may be due to the ability of the nanoparticles (NPs) to be trapped within the silicone matrix and in some polymer chains then, a 3D mesh formation would result in a physical interaction which may lead to an increase in the density of the silicone and the resistance to tearing [2]. This outcome might be caused by an increase in intermolecular pressures and the adsorption of the polymer chain on the filler surface brought on by an increase in filler concentration [10].

The reduction in tear strength and tensile strength at the 1% concentration may be due to the very small amounts of fillers which act as impurities that would affect the polymerization process of the silicone without the formation of a 3D mesh, while the decrease in tear strength at 3%, which is a high concentration group that may cause agglomeration of the fillers within the silicone matrix and results in restriction of flow and movement of polymers matrix when the stretching forces increased [1].

**Figure 4:**
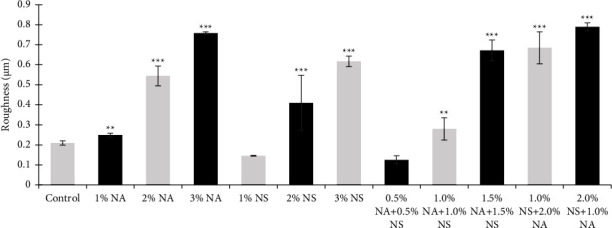
Roughness for different wt. % of nanoalumina (NA), nanosilica (NS), and a hybrid nanoparticle HySA.  ^*∗∗*^*P*  <  0.01,  ^*∗∗∗*^*P*  <  0.001.

Roughness test results showed that the effect of artificial aging for 100 hours after adding nanoparticles was significantly increased with hybrid nanoparticle 2% NA + 1% NS, which was (0.78) compared to the control group (0.20). This is because hybrid nanoparticles of NA and NS, even if these particles were removed, remained closely connected with the polymeric chains under various circumstances. It would be predicted that the polymer's increased porosity would result in increased surface roughness [10] as shown in Figure 4.

Based on the regression analysis, the relationship between the four measured outcomes to the weight percentage of NA and NS can be identified. Hardness and roughness are muchly affected by the weight percentage of NA compared to NS. Similarly, tear strength is muchly reduced with the increase in NA compared to NS. In contrast, tensile is muchly reduced with an increase in NS compared to NA.

The result agreed with a study conducted by Han et al. recommended the incorporation of nano-oxides of Ti, Zn, and Ce (surface treated) at 2 to 2.5% wt into A-2186 silicone elastomer; these concentrations positively enhanced the mechanical properties. Their results indicated that with increased concentration to 3%, the tear strength, and tensile strength decreased. Contrasting with this, the results of the current study indicated that with the use of 2% nanosilica, there was an improvement in the tear strength, tensile strength, shore A hardness, and surface roughness. However, when the concentration was increased to 3%, all the nano-oxides had agglomerated, resulting in a decrease in the mechanical properties of silicone elastomer [18].

The result of the study mentioned above contradicted the results of a study conducted by Tukmachi and Ali that incorporated the nanosilica at concentrations 4 to 6% wt into maxillofacial silicone M-511 HTV. It stated that all nanosilica concentration groups showed a highly significant increase in tear strength and tensile strength compared to the control group. The 5% group showed the highest mean values among other groups. Shore A hardness showed a highly significant increase with all nanosilica concentrations with the increase being directly proportional to filler concentration increase. In comparison to this study, when the concentration was increased to 3%, all the nano-oxides had agglomerated which resulted in a decrease in the mechanical properties of silicone elastomer [19].

This study has limitations that are important to acknowledge. The study had to be conducted using different brands of nanomaterial due to the unavailability of raw materials from the supplier. Additionally, further analysis may also provide more conclusive findings, such as microstructure and morphology analysis using a scanning electron microscope (SEM) and energy dispersive spectrometer (EDS).

Despite the small number of samples applied for each specification of these two nanoparticles, NS oxide filler 99.9% purity, 30–50 nm, and NA oxide filler 99.9% purity, 40–60 nm to a maxillofacial silicone Tech-sil25, were able to improve the mechanical properties following exposure to 100 hours accelerated weathering expecting that the material can produce facial prostheses with more extended service in practice. Therefore, the null hypothesis has been rejected.

## 5. Conclusions

The following conclusions based on the current study's limitations could be stated: the best addition that improved the mechanical properties was the 2% nanosilica (NS) group over all other groups. The most suitable percentages of the composite hybrid nanoparticle for both nanosilica and nanoalumina fillers were found to be 1% NA + 1% NS, according to this study that enhanced tear strength and tensile strength value while maintaining acceptable values of the hardness and roughness properties for room temperature vulcanized silicone (RTV) Tech-sil25 maxillofacial silicone. This study revealed that adding various types of fillers with different properties and size scales in appropriate proportions to silicone elastomer could enhance some of the mechanical properties. The composite of different types of fillers reinforcement is promising to improve the antiaging properties of the maxillofacial silicone elastomer and maintain some of the mechanical properties (Shore A hardness, tensile strength, tear strength, and roughness), which will provide a clinical advantage to the marginal integrity of a facial prosthesis. That will enhance the esthetic quality of a facial prosthesis by permitting a thinner margin with a better possibility of stretching and less tearing in the prosthesis, which will increase prosthesis service life. Future work should be planned to evaluate the effect of the addition of nanosilica (NS), nanoalumina (NA), and a hybrid nanoparticle (HySA) using additional methodologies like SEM and EDS analysis [20].

## Figures and Tables

**Table 1:** The amount of silicone base (part A) mixed with either wt.% of nanosilica (NS) or wt.% of nanoalumina (NA) or a combination of nanosilica and nanoalumina (NSA) and catalyst (part B) for Tech-sil25 silicone material.

Group	Base (g)	Catalyst (g)	Nanosilica (g)	Nanoalumina (g)
NS 0%	100	11	0	0
NA 0%				

NS 0%	99	11	0	1
NA 1%				

NS 0%	98	10.8	0	2
NA 2%				

NS 0%	97	10.7	0	3
NA 3%				

NS 1%	99	11	1	0
NA 0%				

NS 2%	98	10.8	2	0
NA 0%				

NS 3%	97	10.7	3	0
NA 0%				

NS 0.5%	99	11	0.5	0.5
NA 0.5%				

NS 1%	98	10.8	1	1
NA 1%				

NS 1.5%	97	10.7	1.5	1.5
NA 1.5%				

NS 1%	97	10.7	1	2
NA 2%				

NS 2%	97	10.7	1	2
NA 1%				

**Table 2:** The comparison of 1%, 2%, and 3% wt. nanoalumina (NA) addition on Tech-sil25 maxillofacial silicone mechanical properties.

	Weight percent (wt. %)	Values mean ± SD	*F* statistic^a^ (*df*)	*P* value^a^
Shore A hardness (mm)	Control	27.00 ± 1.00	30.15	
	1% NA	27.33 ± 2.08	(3; 8)	0.000^b^
	2% NA	30.67 ± 0.58		
	3% NA	36.67 ± 1.53*∗*		

Tensile (MPa)	Control	3.82 ± 0.59	13.46	0.002^c^
	1% NA	4.79 ± 0.15	(3; 8)	
	2% NA	5.56 ± 0.37*∗*		
	3% NA	3.63 ± 0.45		

Tear (kN/m)	Control	24.06 ± 0.09	97.77	0.000^b^
	1% NA	24.57 ± 0.30	(3; 8)	
	2% NA	25.65 ± 0.08*∗*		
	3% NA	23.13 ± 0.17		

Roughness (*μ*m)	Control	0.21 ± 0.01	300.44	0.000^b^
	1% NA	0.25 ± 0.01	(3; 8)	
	2% NA	0.54 ± 0.04		
	3% NA	0.76 ± 0.01*∗*		

^*a*^One-way ANOVA test. ^*b*^ All 3 pairs of mean scores are significantly different by post hoc test (Scheffe procedure). ^*c*^ All 2 pairs of mean scores are significantly different by post hoc test (Scheffe procedure). ^∗^indicates the highest mean value.

**Table 3:** The comparison of 1%, 2%, and 3% wt. nanosilica (NS) addition on Tech-sil25 maxillofacial silicone mechanical properties.

	Weight percent (wt. %)	Values mean ± SD	*F* statistic ^*a*^ (*df*)	*P* value ^*a*^
Shore A hardness (mm)	Control	27.00 ± 1.00	45.27	
	1% NS	29.33 ± 1.15	(3; 8)	0.000^*b*^
	2% NS	33.66 ± 1.52		
	3% NS	38.66 ± 1.52*∗*		

Tensile (MPa)	Control	3.82 ± 0.59	13.46	
	1% NS	5.16 ± 0.34	(3; 8)	0.002^*c*^
	2% NS	6.20 ± 0.31*∗*		
	3% NS	4.24 ± 0.59		

Tear (kN/m)	Control	24.06 ± 0.09	84.08	
	1% NS	24.88 ± 0.05	(3; 8)	0.000^*b*^
	2% NS	25.89 ± 0.07*∗*		
	3% NS	22.60 ± 0.50		

Roughness (*μ*m)	Control	0.20 ± 0.01	27.61	
	1% NS	0.14 ± 0.02	(3; 8)	0.000^*b*^
	2% NS	0.40 ± 0.13		
	3% NS	0.61 ± 0.02*∗*		

^*a*^One-way ANOVA test. ^*b*^ All 3 pairs of mean scores are significantly different by post hoc test (Scheffe procedure). ^*c*^ All 2 pairs of mean scores are significantly different by post hoc test (Scheffe procedure). ^∗^indicates the highest mean value.

**Table 4:** The comparison of addition a combination (NSA) on Tech-sil25 maxillofacial silicone mechanical properties.

	Weight percent (wt. %)	Values mean ± SD	*F* statistic ^*a*^ (*df*)	*P* value ^*a*^
Shore A hardness (mm)	Control	27.0 ± 1.00	47.02	
	NA 0.5% + NS 0.5%	26.6 ± 5.57	(3; 8)	0.000^*b*^
	NA 1% + NS 1%	30.3 ± 1.52		
	NA 1.5% + NS 1.5%	35.3 ± 1.15		
	NA 2% + NS 1%	35.3 ± 0.57		
	NA 1% + NS 2%	36.6 ± 1.52*∗*		

Tensile (MPa)	Control	3.82 ± 0.59	26.39	
	NA 0.5% + NS 0.5%	4.91 ± 0.04	(3; 8)	0.000^*b*^
	NA 1% + NS 1%	5.95 ± 0.25*∗*		
	NA 1.5% + NS 1.5%	3.65 ± 0.09		
	NA 2% + NS 1%	3.43 ± 0.10		
	NA 1% + NS 2%	3.80 ± 0.45		

Tear (kN/m)	Control	24.0 ± 0.09	79.12	
	NA 0.5% + NS 0.5%	24.7 ± 0.13	(3; 8)	0.000^*b*^
	NA 1% + NS 1%	25.8 ± 0.06*∗*		
	NA 1.5% + NS 1.5%	22.8 ± 0.08		
	NA 2% + NS 1%	23.2 ± 0.07		
	NA 1% + NS 2%	22.8 ± 0.52		

Roughness (*μ*m)	Control	0.20 ± 0.01	112.6	
	NA 0.5% + NS 0.5%	0.12 ± 0.02	(3; 8)	0.000^*b*^
	NA 1% + NS 1%	0.28 ± 0.05		
	NA 1.5% + NS 1.5%	0.67 ± 0.05		
	NA 2% + NS 1%	0.68 ± 0.01		
	NA 1% + NS 2%	0.78 ± 0.07*∗*		

^*a*^One-way ANOVA test. ^*b*^ All 3 pairs of mean scores are significantly different by post hoc test (Scheffe procedure). ^∗^indicates the highest mean value.

## Data Availability

The research data used to support the findings of the current study are available from the corresponding author upon request.
